# Managing health professional migration from sub-Saharan Africa to Canada: a stakeholder inquiry into policy options

**DOI:** 10.1186/1478-4491-4-22

**Published:** 2006-08-14

**Authors:** Ronald Labonté, Corinne Packer, Nathan Klassen

**Affiliations:** 1 Canada Research Chair Globalization/Health Equity, Institute of Population Health & Department of Epidemiology and Community Medicine, University of Ottawa, Ottawa, Ontario, Canada; 2 Institute of Population Health, University of Ottawa, Ottawa, Canada; 3 Saskatchewan Population Health and Evaluation Research Unit, University of Regina, Regina, Canada

## Abstract

**Background:**

Canada is a major recipient of foreign-trained health professionals, notably physicians from South Africa and other sub-Saharan African countries. Nurse migration from these countries, while comparatively small, is rising. African countries, meanwhile, have a critical shortage of professionals and a disproportionate burden of disease. What policy options could Canada pursue that balanced the right to health of Africans losing their health workers with the right of these workers to seek migration to countries such as Canada?

**Methods:**

We interviewed a small sample of émigré South African physicians (n = 7) and a larger purposive sample of representatives of Canadian federal, provincial, regional and health professional departments/organizations (n = 25); conducted a policy colloquium with stakeholder organizations (n = 21); and undertook new analyses of secondary data to determine recent trends in health human resource flows between sub-Saharan Africa and Canada.

**Results:**

Flows from sub-Saharan Africa to Canada have increased since the early 1990s, although they may now have peaked for physicians from South Africa. Reasons given for this flow are consistent with other studies of push/pull factors. Of 8 different policy options presented to study participants, only one received unanimous strong support (increasing domestic self-sufficiency), one other received strong support (increased health system strengthening in source country), two others mixed support (voluntary codes on ethical recruitment, bilateral or multilateral agreements to manage flows) and four others little support or complete rejection (increased training of auxiliary health workers in Africa ineligible for licensing in Canada, bonding, reparation payments for training-cost losses and restrictions on immigration of health professionals from critically underserved countries).

**Conclusion:**

Reducing pull factors by improving domestic supply and reducing push factors by strengthening source country health systems have the greatest policy traction in Canada. The latter, however, is not perceived as presently high on Canadian stakeholder organizations' policy agendas, although support for it could grow if it is promoted. Canada is not seen as "actively' recruiting" ("poaching") health workers from developing countries. Recent changes in immigration policy, ongoing advertising in southern African journals and promotion of migration by private agencies, however, blurs the distinction between active and passive recruitment.

## Background

The International Organization for Migration (IOM) estimates that about 20 000 Africans leave Africa every year to take up employment in industrialized countries. While we do not know how many of these are health professionals – largely because of inadequate systems for gathering such statistics in African countries – two different studies found that a quarter to two thirds of health workers interviewed in Africa intended to migrate [1,2].

According to the most conservative estimates, more than 7% of nurses [3] and 22% of physicians [4] in Canada are foreign-trained. While Canada is more dependent on foreign-trained health professionals than most other OECD nations, it places slightly lower than some other English-speaking OECD countries, such as the United Kingdom, the United States of America, Australia and New Zealand [5].

Canada nonetheless has a comparatively higher proportion of foreign-trained physicians from sub-Saharan Africa (SSA) [6]; notably of 12 OECD countries surveyed Canada had the highest proportion of South African-trained physicians, accounting for nearly 10% of Canada's total population of foreign-trained physicians. The United Kingdom was next, with 7% [5]. Between 1986 and 2000 (the last year for which comparative data are available) the number of physicians migrating to Canada from South Africa alone outnumbered those coming from the United Kingdom/Ireland, India and the entire regions of Europe and Asia [7].

In light of Canada's dependence on foreign-trained health workers and the growing international concern for the losses such migration creates for many SSA countries, we conducted a study to ascertain recent trends on health human resource (HHR) flows, perceived reasons for such flows, and key Canadian stakeholder awareness of, and support for, options by which Canada might help mitigate the negative effects of HHR migration from this region.

## Methods

The findings presented in this article derive from semi-structured interviews conducted with a purposive sample of leaders – individuals with decision- and policy-making influence and authority – from 25 key Canadian stakeholder organizations, including physician and nursing colleges (n = 13), pertinent federal departments (n = 5) and provincial health ministries and regional health authorities (n = 7). In the case of the latter two, a purposive sample of provinces with high percentages of health professionals from developing countries was selected. Interim findings were presented in a multi-stakeholder colloquium to enrich an understanding of why some policy options received support while others did not and to examine issues in policy implementation (n = 21). Special tabulations of existing data sets on foreign-trained health professionals in Canada were also performed, and interviews with a small convenience sample (n = 7) of émigré South African physicians practising in Canada were conducted.

## Results and discussion

We first describe some of the dynamics in the recent flows of physicians and nurses from SSA to Canada, and then turn to our findings on policy options. We conclude with a brief commentary on these options, incorporating information not available at the time of the interviews or considered in this first phase of our ongoing programme of HHR migration research.

### SSA-trained physicians in Canada

**Figure 1:**
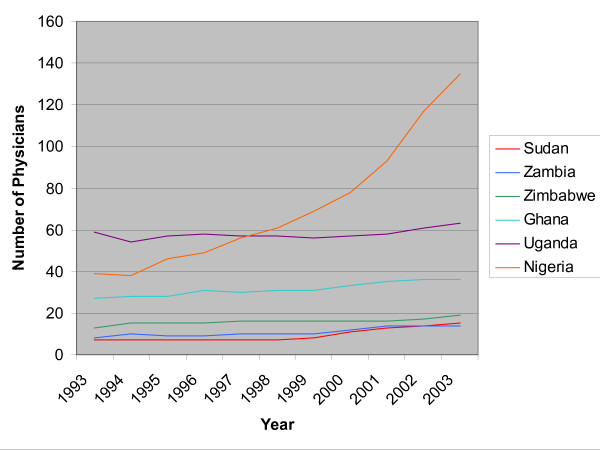
Number of physicians from principal SSA source countries practising in Canada, 1993–2003.

**Figure 2:**
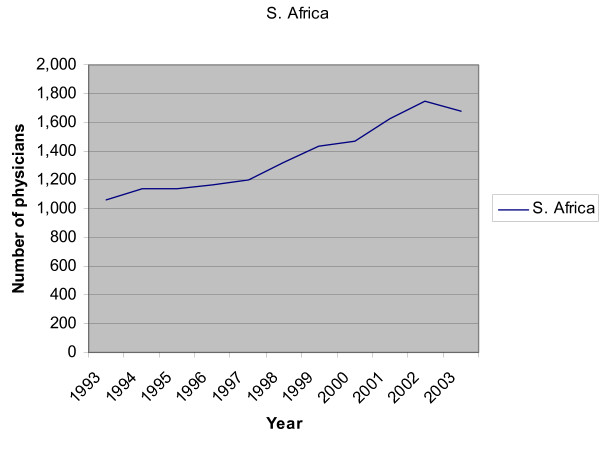
Number of South African physicians practising in Canada, 1993–2003.

Although the number of foreign-trained physicians working in Canada has remained constant over the last four decades, the number coming from developing countries, many of which themselves are facing severe physician shortages, has risen steeply. Of greatest concern are increases in physicians migrating to Canada from Nigeria and South Africa: The number of South African-trained physicians practising in Canada has risen over 60% in the last decade, while the number of physicians trained in Nigeria and now working in Canada has more than tripled in the same period. In the case of Uganda, with a meagre number of physicians (a total of 2209), it means that 3% of its physicians, trained at its own expense, are now working in Canada alone (see 2004 statistics in WHO's Global Atlas on Health Human Resources at ). In the case of South Africa, nearly 5% of its physicians are now working in Canada. While South Africa has many more physicians in proportion to its population than Uganda, it is also a significant source country of physicians to other English-speaking OECD countries in addition to Canada. Figures 1 and 2 illustrate the ten principal SSA source countries of foreign-trained physicians in Canada.

The provincial breakdown provided in Table 1 demonstrates a tendency of SSA-trained physicians in Canada to locate (or be located) as cohorts with members of their own community, ethnic background or country of origin. Respondents attributed this to classmates, friends and colleagues following those already established in Canada.

**Table 1:** Provincial breakdown of physicians having graduated from selected sub-Saharan African countries, 2003

	**Alberta**	**British Columbia**	**Manitoba**	**New Brunswick**	**Newfoundland**	**Northwest Territories**
**Ghana**	1	1	3	0	3	0
**Nigeria**	17	7	6	4	21	0
**South Africa**	373	592	134	8	50	2
**Tanzania**	3	0	0	0	0	0
**Uganda**	7	12	2	0	5	0
**Zambia**	5	4	0	0	0	0
**Zimbabwe**	2	8	0	1	1	0
						

	**Nova Scotia**	**Nunavut**	**Ontario**	**Prince Edward Island**	**Quebec**	**Saskatchewan**

**Ghana**	1	0	21	0	0	6
**Nigeria**	6	0	47	0	1	23
**South Africa**	22	1	336	2	13	266
**Tanzania**	0	0	4	0	0	0
**Uganda**	1	0	30	0	0	6
**Zambia**	0	0	2	0	1	1
**Zimbabwe**	0	0	5	0	0	2

Source: CIHI, Southam Medical Database. Statistics gathered at special request of authors and issued by CIHI on 12 August 2005. Table excludes the Yukon Territory, which has no physicians from these countries.

It is also likely that recruiters contribute to this formation, hiring graduates from certain SSA universities or hospitals that they believe produce highly qualified physicians. A few of our respondents indicated this preference for certain training schools in SSA; one lamented that it was clear from recent recruits from the training schools it had regularly dealt with that the quality of physicians produced had deteriorated. As another example, fully 79% of all SSA physicians working in the United States were trained at only 10 medical schools, most of these located in South Africa and Nigeria, i.e. Canada's two top SSA source countries [8].

The strong command of the English language, recognized quality of training and years of experience with which they come also gives them natural advantages, compared to foreign-trained physicians from non-English-speaking countries.

The majority of foreign-trained graduates practise family medicine [9]. This does not necessarily mean that all are trained in family medicine, as the greater majority come to Canada with specialized training and experience but are only, or more easily, able to find work in family medicine. Domestically-trained physicians (i.e. physicians trained in Canadian universities) entering family practice became in short supply in the 1990s, owing in part to a change in Canadian policy to increase specialty residency positions relative to family medicine [10].

Replicating a global trend, foreign-trained physicians also are more likely to work in rural areas of Canada, where fewer domestically-trained physicians are willing to work and demand for family practitioners is high. In 2004 26.3% of all physicians in rural Canada were foreign-educated [11]. This is underscored in Table 2, which shows variations in the provincial and territorial reliance upon foreign-trained physicians, with smaller, more rural areas showing the greatest dependency. One exception to this trend is Quebec, which at 10.8% has the lowest percentage of foreign-trained physicians. The pool of French-speaking health professionals potentially recruited or attracted to Quebec, a French-speaking province, would be comparatively smaller than the pool for English-speaking provinces. The precise reasons for the lower percentage of foreign-trained physicians in Quebec, however, are not known.

**Table 2:** Number of Canadian-educated and foreign-educated physicians by province/territory, Canada, 2004

	**Foreign-trained**	**Domestically-trained**	**% foreign-trained**
Newfoundland and Labrador	393	557	41%
Prince Edward Island	33	165	17%
Nova Scotia	560	1432	28%
New Brunswick	281	976	22%
Quebec	1758	14 366	11%
Ontario	5272	16 783	24%
Manitoba	537	1369	28%
Saskatchewan	793	728	52%
Alberta	1549	4350	26%
British Columbia	2244	6008	27%
Yukon Territory	17	34	33%
Northwest Territories	13	38	25%
Nunavut	2	5	29%

**Canada total**	**13 453**	**46 811**	**22%**

Source: CIHI, Southam Medical Database. Supply, Distribution and Migration of Canadian Physicians, 2004, Ottawa, CIHI, 2005, pp. 35–48. Available at . Figures represent physicians in family medicine and specialists.

### SSA-trained nurses in Canada

The number of foreign-trained nurses in Canada has remained constant over the last five years [12]. The proportion of foreign-trained registered nurses (RNs) in Canada is considerably less than that of foreign-trained physicians, representing only 7.4% of the total registered nurses in Canada [13]. The number of internationally-educated nurses (registered, licensed practical and registered psychiatric) applying for registration in Canada, however, increased significantly from 1792 in 1999 to 4546 in 2003 [13]. The province of British Columbia (BC) has the highest proportion of all foreign-trained nurses, whereas the province of Ontario has the greatest proportion of African-trained nurses. Nearly half of all foreign-trained RNs come from either the Philippines (approximately 30%) or the United Kingdom (just over 20%) [13]. SSA is not presently a significant source of nurses to Canada, but trends demonstrating a slow but steady increase in numbers from the region cannot be ignored.

**Figure 3:**
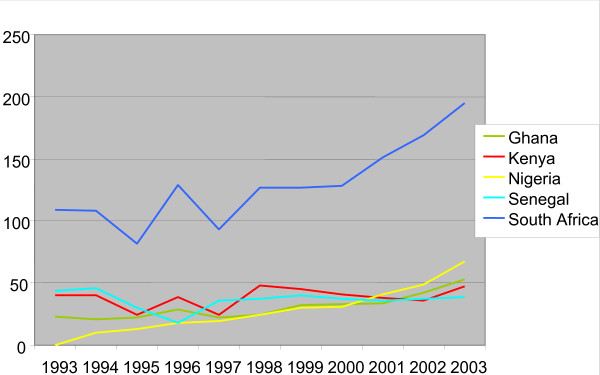
Number of registered nurses from principal SSA source countries practising in Canada, 1993–2003.

A survey of employed registered nurses in Canada who had graduated from a SSA country found that they came from only nine (of 53) SSA countries. In 2003, 524 nurses in Canada were trained in SSA, more than 25% of these in South Africa. Figure 3 demonstrates the upward trend in the number of SSA-trained RNs in the Canadian nursing workforce over the last decade. Only countries with 20 or more nationals are represented in the figure. Ethiopia joined these ranks only in 2003, when it saw 20 of its nurses working in Canada that year. Like their physician counterparts, South African nurses represent the overwhelming majority of SSA-trained nurses now practising in Canada, with nearly three times more nurses than the next most represented group, from Nigeria. As noted with their physician counterparts, the large increase in Nigerian-trained nurses practising in Canada (from 0 to 67 over one decade) is alarming. Also noteworthy is the doubling – in one decade – in the number of Ghanaian-trained nurses coming to work in Canada.

Table 3 shows the distribution of SSA-trained nurses throughout Canada in 2003. Only provinces with five or more SSA-trained nurses are listed. The table suggests that, as with physicians, nurses from specific countries tend to congregate in the same provinces. With the exception of Senegalese-trained nurses congregating in Quebec for linguistic reasons, it is not possible to establish whether this phenomenon is the result of targeted recruitment or of simple word-of-mouth from nationals based in Canada. No nursing colleges and associations interviewed claimed knowledge of active or targeted recruitment, lending plausibility, though not certainty, to the latter hypothesis of nurses self-locating.

**Table 3:** Number of employed registered nurses by African country of graduation and province, 2003*

	**Quebec**	**Ontario**	**Manitoba**	**Saskatchewan**	**Alberta**	**British Columbia**
Dem. Rep. of Congo	9	5				
Zimbabwe		7				
Ethiopia		22				
Ghana		47				6
Kenya		24			11	12
Nigeria		42				7
Senegal	39					
Somalia		11				
South Africa		76	8	11	21	79
Total*	67	256	18	18	42	112

* Including nurses from SSA countries with fewer than five nurses in any province or territory. Source: CIHI. Statistics gathered at special request of authors and issued by CIHI on 12 August 2005.

The figures of health professional migration cited in this article underestimate the actual flows from source countries to Canada, since the data sets refer only to licensed or registered foreign-trained professionals. Other studies suggest that large numbers of foreign-trained physicians and nurses fail to qualify for practice in Canada; one estimate for nurses found that approximately two thirds of foreign-trained nurses who began the process of licensure in Canada failed to complete it for a number of reasons [14]. This "brain waste" is the obverse of "brain drain", and fully using the trained health professionals who are unemployed in both source and recipient countries could go some distance towards mitigating the present "push"/"pull" dynamics underpinning global HHR migration.

### Is domestic demand driving immigration?

A significant portion of our analysis was devoted to understanding the factors driving the demand for SSA health professionals within Canada. While our focus in this article is on the "pull" factors as such, this should not be construed as diminishing the significant role of "push" factors in source countries in the brain drain phenomenon, which many of our respondents identified as the principal reasons for HHR migration from many developing countries. Table 4 cites the principal "push/pull" factors cited by our respondents, which affirms much of what is already known about these dynamics (the only slightly novel items are reference to gender discrimination/violence as push factors, and the tolerant, multi-ethnic nature of Canadian society).

**Table 4:** Summary of push and pull factors identified by respondents

**Push factors**	**Pull factors**
**Job security**	

No jobs available	Jobs available; colleagues, friends and recruiters telling them about opportunities
Lack of promotions	
Risk of losing jobs due to lack of funds	

**Working conditions**	

Low salaries	Reasonable remuneration – able to save money
Non-payment of salaries (non-payment of housing allowance)	
Deteriorating work environment/facilities	
Inadequate medicine and equipment	
Significant stress, overtime and generally poor conditions of service resulting in fatigue and burn-out	Regular workload
Inability to treat patients due to poor services and medicine	
Impossible patient-health care provider ratios, making it difficult to give quality care	Reasonable conditions of work
Poor health human resources planning	

**Economic and political considerations**	

Disarray in severely economically depressed SSA countries	Canada a wealthy, democratic country
Political and racial upheaval	Not corrupt
Gender discrimination	

**Physical security**	

Carjackings	Safe country
Muggings	
Significant criminality	
Gender-based violence	
Significant exposure to HIV – risk of infection through treatment of patients	

**Quality of life**	

Poor accommodation	Canada tolerant, multi-ethnic
Lack of transport to go to work	Good quality of life
Inability to live a decent life	

**Education**	

Diminishing quality of education for children	Greater opportunities for children – good education and ability to earn a decent living

The primary factors driving demand for foreign-trained physicians and nurses in Canada are the demographic shift occurring in the country, poor HHR planning, rural to urban and interprovincial drains within Canada, and regional competition for HHR from the United States.

Canada, as much of the developed world, is undergoing a dramatic demographic shift. The dual challenge of an ageing population and declining birth rates during the latter half of the 20th century places a significant burden upon Canada's health system: "Elderly patients use more health services, and a growing proportion of patients are elderly" [10]. According to one estimate, patients aged 65 years and over consume approximately 70% of the Canadian health care budget [15]. The ageing population poses another challenge: the very professionals who are needed to build capacity to meet their increasing demand themselves are nearing retirement [9,13,14]. There are fewer physicians aged 35 and under and more aged 65 and over [10].

It is not only the ageing but also the more feminized workforce that is leaving Canadians with a supply shortage. More and more women are entering medicine in Canada. Female physicians work less than their male counterparts, particularly during the child-rearing ages of 35 to 44. The Canadian physician workforce therefore has more women and elderly physicians who tend to work less, while the number of ageing Canadians and the Canadian population in general continues to grow [10].

Complicating health system planning is Canada's federal structure. Constitutional responsibility for the delivery of health services falls to the provinces. However, the federal government's control over transfer payments to the provinces and the federal *Canada Health Act*, which places restrictions on provincial health systems in order to qualify for such transfers, creates a highly politicized environment for health policy-making. In addition, most provinces have moved towards a regional model for the delivery of health services, often referred to as regional health authorities (RHAs). This fragmentation of authority and responsibility enhances the complexity of HHR planning within Canada and ensures that quick policy responses are improbable.

The jurisdictional split exacerbates two of the greatest challenges to HHR planning: the rural to urban and interprovincial brain drains. Rural depopulation and increasing urbanization in much of Canada (as in many other developed and developing countries) has made retention of rural health workers (notably physicians) very difficult. Urban centres often offer better working conditions and facilities and educational, employment and sociocultural opportunities that are simply not available in rural areas.

Many initiatives have sprung up to address this challenge, e.g. telemedicine to reduce the professional isolation, financial incentives for service in rural areas, establishment of rural medical schools and the direct recruitment of foreign-trained physicians into rural vacancies. Nonetheless, it is the relative lack of rural health professionals that is the major "pull" to recruit foreign-trained workers.

The interprovincial drain can be thought of in much the same terms as the rural-to-urban drain. Some provinces have better climate/weather, greater wealth (increased earning potential) and more practice opportunities (enhanced career satisfaction), all of which may be attractive to health professionals in other parts of the country. The result is that health professionals are drained from the provinces that have the hardest time retaining staff to the provinces that do not, often from predominantly rural and poorer provinces to predominantly urban, or highly urbanized, wealthier provinces. These professionals are difficult to replace.

In addition, Canada's aggregate HHR shortage places the provinces in competition, rather than cooperation, over recruiting and retaining experienced and newly graduated physicians. Yet even the more attractive jurisdictions are facing acute HHR shortages; the College of Physician and Surgeons in British Columbia (in terms of climate, the most popular province in Canada) has stated that the province "will continue to rely on physician immigration in the foreseeable future" to meet its needs [16].

Canada faces another challenge due to its proximity to the United States. The USA is facing the same demographic shift as Canada, but the needs of its much larger population are far greater. The shortfall of physicians in the United States is expected to be between 85 000 and 200 000 by the year 2020 [17]. The shortfall of nurses is expected to be 400 000 to 700 000 by the year 2020 [18]. American health providers frequently hold employment fairs and pursue other recruitment initiatives to lure Canadian medical professionals to the United States. While the drain to the USA has been slowing in recent years, it is clear that Canada will face increasingly aggressive competition for its health human resources in the very near future [28].

Another measure that increases the "pull" for foreign-trained health care professionals is Citizenship and Immigration Canada's points system, adopted in 2002. The points system, which accords individuals with advanced education, work experience and English and French linguistic abilities more points towards qualification for immigration, biases towards highly trained professionals, especially those from English-speaking Commonwealth countries.

A related and lesser known measure is the Provincial Nominee Program (PNP). PNPs are individually negotiated immigration agreements between provinces and the federal government. These agreements allow persons who could help reduce labour shortages specified by the provinces to "fast-track" through the immigration process. To date, nine of Canada's provinces have implemented PNPs, two of which have created a special stream for health professionals.

### Shortages in the domestic supply of physicians and nurses

Appropriate HHR planning within Canada could have mitigated many of the effects of this demographic shift. Policy-makers since the 1980s, however, have oscillated between believing in either an oversupply of physicians or a shortage. A series of policy recommendations, first in 1980 and then 1984, recommended a reduction in the number of medical students entering the system. Neither of these policies was implemented [10]. In 1991 the Barer-Stoddart Report recommended, among many options, a "10% reduction in medical students but [emphasized a need to] maintain physician to population ratios" [10]. The reduction was implemented beginning with the class of 1997, but policy-makers failed to ensure that physician-to-population ratios remained constant (Barer-Stoddert recommended 1.9 physicians per 1000 citizens) [10]. This policy and others, such as the decision to eliminate rotating internships, to reduce and restrict the number of retraining opportunities and to increase the number of specialty residency positions relative to family medicine, have contributed to a decreased inflow of physicians [10].

The number of registered nurses (RNs) employed in Canada has risen only slightly over the last five years, after a decade of relative stagnation [19]; the number of nurses has risen only 5% in the last 15 years, while Canada's population has increased by nearly 9% (statistics available at ). This has had a damaging impact overall on our health care system [13].

There were 9.8 nurses per 1000 in Canada in 2003, a higher figure than the average of 8.2 in OECD countries. Most other OECD countries, however, have had greater increases in nurses in recent years [35]; a 2004 OECD study reported that Canada had the highest relative nursing shortage of the six countries examined, at 6.9% of the present workforce [21].

Moreover, the slight gains experienced in Canada likely will be short-lived, if not already lost. In the early 1990s, cuts to the Canadian health care budget led to the elimination of nursing positions in several provinces. Over the last decade there was a reduction of over 50% in the number of seats in Canadian nursing schools [22]. This is largely attributable to changes in all RN diploma programmes to baccalaureate degree training in the last decade. This has resulted in a decrease, at least in the short term, in the number of graduates from RN programmes and the number entering the workforce [13].

The reduction in the supply of nurses is further compounded by an ageing workforce, as noted earlier. Retention rates in many Canadian nursing schools are also too low, resulting in wastage of school resources and "unnecessarily [reducing] the number of potential nurses in the health care system" [19]. Low rates of nurse retention in the workforce further erode any training gains.

Financial constraints in nursing schools limit their resources, in particular their ability to hire new faculty or provide back-up in the absence of teaching staff. Unless substantial financial resources are provided, there is a limited capacity to increase enrolments in nursing schools. Once again, shortages in the stock of Canadian nurses will beg to be filled in some manner, foreign-trained nurses providing the easiest option.

### Findings on policy options

There is little disagreement about the nature of the crisis in global health human resources, exacerbated by the flow of HHRs from developing to developed countries. The seriousness of this problem is reflected in its becoming the theme of the 2006 *World health report *[23] and World Health Day. But little international agreement has been reached on what can be done to remedy it, in terms of what is politically feasible and who should assume responsibility, since the issue first became a multilateral concern in the 1970s. The difficulty in achieving consensus on policy solutions also became apparent in our analysis of key informant opinions (and that of the organizations they represented) of eight possible options to ensure that HHR migration did not come at the health expense of SSA countries. The results are summarized below.

#### Codes of practice for ethical recruitment

None of the organizations interviewed had adopted a code of practice for ethical recruitment, although a small number had issued statements against the active recruitment of health care professionals from developing countries with acute shortages of practitioners. Indeed, very few concerned organizations had formally acknowledged the extent to which Canada depends on foreign-trained health care professionals to sustain the Canadian health care system or that it poses a problem to developing countries.

Mention of the *Commonwealth Code of Practice for the International Recruitment of Health Workers *was made by a few respondents. Respondents cited the Commonwealth Code as a positive measure, although they stressed its voluntary (non-binding) character. One informant made reference to the *Melbourne Manifesto*, a code of practice for the international recruitment of health care professionals adopted by delegates to the World Organization of National Colleges, Academies and Academic Associations of General Practitioners/Family Physicians (WONCA) meeting in Melbourne, Australia, on 3 May 2002. In comparative terms, the *Melbourne Manifesto *is more specific in its guidelines and therefore potentially more useful as a tool than is the Commonwealth Code. Judging by absence of reference, neither the *Code *nor the *Manifesto *was well known or considered seriously by most informants and thus they have yet to make any significant impact in Canada.

Yet when interview participants were asked whether they would support the adoption of a code of practice for ethical recruitment, responses were generally positive. Respondents qualified that the codes should be voluntary, even while acknowledging that such codes have not worked out because of their voluntary nature. Both interview and colloquium participants expressed concern that mandatory codes were unlikely to be ratified, while a small number noted that proper enforcement of such a code would require significant funding.

Since the interviews for our survey were held, new principles commonly referred to as the "London Declaration" (April 2005) were formulated and endorsed at an international conference on the global health workforce [24]. The conference agreed on the following four key points:

• All countries must strive to attain self-sufficiency in their health care workforce without generating adverse consequences for other countries.

• Developed countries must assist developing countries to expand their capacity to train and retain physicians and nurses, to enable them to become self-sufficient.

• All countries must ensure that their health care workers are educated, funded and supported to meet the health care needs of their populations.

• Action to combat the skills drain in this area must balance the right to health of populations and other individual human rights.

This is one of the most explicit references to the human rights dimension of global HHR migration, a point we return to briefly in our conclusion.

#### Improved health human resources planning in Canada

All participants interviewed felt strongly that better HHR planning in receiving countries is a top priority; many indicated that their organizations were actively pursuing this aim. Respondents felt that ensuring an adequate stock of Canadian-trained health care professionals, by reducing the "pull" factor of available positions, would be the best way to reduce the inflow of foreign-trained health care professionals from developing countries. Respondents cited two principal strategies by which Canada might improve HHR planning and management.

##### • Increase enrolments in Canada

The general consensus was that Canada should increase the number of training spots in schools of medicine and nursing. One respondent cited an average of five to nine applicants for every medical spot in Canada, resulting in wasted potential for self-sustainability or an ensured steady stock. Respondents complained that Canada is not doing a good job predicting and planning its resource needs. One noted: "Every ten years we say we have too many and we close down enrolments and then every five years thereafter we say we have too few and open up enrolments again and there's never a proper fit."

Most provinces have already reacted to the shortage by making substantial increases in medical and nursing school enrolments. However, the increases are not enough to meet demand, especially in light of anticipated recruitment of Canadian health professionals by health systems in the United States, and are financially costly and difficult to sustain. The Canadian Medical Association (CMA), in collaboration with a number of other medical professional groups, is calling on Canadian governments to create a $CAD1 billion "health human resources reinvestment fund" to ensure an adequate supply of domestically trained health care professionals. The CMA president is critical that "we don't have a policy that Canada should be self-sustaining...we're poaching from other jurisdictions" [25].

##### • License foreign-trained health professionals already in Canada but unable to practice

The priority target group mentioned by a number of respondents and in various reports by provincial and national medical associations are non-nationals who have obtained their degree abroad, have immigrated to Canada but who have difficulty obtaining certification in Canada. Respondents encouraged the creation of mechanisms to assist these individuals to meet Canadian standards. The point was also made that it is not always medical upgrading that is required, but enhanced language training.

Another group of similar concern consists of Canadians who have obtained their training outside Canada. The College of Physicians & Surgeons of Ontario (CPSO) reports that, in Ontario alone, approximately 200 Ontarians graduate each year from medical schools outside Canada. Roughly 100 Canadians are enrolled each year in four schools of medicine in Ireland alone [26], partly a reflection of inadequate training spaces in Canadian medical schools. Fifteen percent of the 1929 medical doctors entering into their first year of residency in Canada in the 2004–2005 school year were Canadian citizens or permanent residents who obtained their MD training abroad [27]. These are the lucky ones able to obtain residencies. Some respondents called for improved mechanisms to evaluate degrees obtained by Canadians abroad and, in the event of inadequate equivalencies, to make residency and upgrading available so that they can meet Canadian standards. The CPSO has similarly issued recommendations urging the government to assess the qualifications of all foreign-trained medical graduates, significantly expand training opportunities and eliminate existing barriers [28].

#### Multilateral/bilateral agreements

The New Partnership for Africa's Development (NEPAD) health strategy specifically refers to the necessity of developing some form of bi- or multilateral agreement to manage the brain drain of professionals from African countries to the benefit of source countries [29]. Agreements would entail measures for managed migration so that there would be no or little net loss to source countries (see Table 5 for a variety of options identified by respondents, or in the literature, for what such agreements might include).

**Table 5:** Examples of specific contents of bilateral/multilateral agreements

• receiving country financing of overtraining in source country;
• enhanced aid that exceeds estimates of loss and is separated from commitments made to help countries achieve the Millennium Development Goals;
• bilateral tax agreements allowing émigrés to remit taxes to source countries;
• taxes on remittances tithed to health professional training or health system financing;
• financial or other forms of encouragement for émigré health professionals to return after a certain period;
• facilitated two-way staff flows to reduce strain on source countries.

Respondents felt that, if such agreements were to take place, they would more likely be in the form of Memoranda of Understanding (MoUs). But respondents were also generally ambivalent towards this option, foreseeing numerous challenges. Most felt it would require a lot of organization and would give rise to many practical problems, the most evident being the monitoring of the agreements. Such agreements typically provide for the return of health professionals to the source country after two to three years abroad, leading other respondents to anticipate problems stemming from health professionals' refusing to return once established with their families in Canada. This would appear to be a valid concern, given that foreign-trained health professionals in Canada tend to be older upon migration, and therefore more likely to have children. This was corroborated by respondents, who consistently described foreign-trained health care professionals coming to Canada as older and with young families. This supports our own study findings that better educational opportunities for children in Canada present a significant pull factor for migration.

Others pointed out that other countries could simply "free-ride" on any bilateral agreements, since bilateral agreements would result in less competition from typical receiving countries or financial support to overtrain in sending countries, enlarging the pool of potential émigré health professionals. This possibility raised the prospect of developing multilateral agreements, such as a multilateral framework convention, which respondents felt would be particularly long in the making, complicated and with uncertain results.

One respondent stressed that multilateral agreements would be near-to-impossible to monitor and oversee, but that bilateral agreements could work with the right framework behind them. Others stated no opinion on this option, as they had either never considered it or found it not to fall within their mandate. Some felt such agreements are not needed and that they would be a waste of time, effort and resources. One respondent flatly observed: "We live in a global market and professionals are free to move. It is up to the developing countries to set their own policies on how to deal with them." This sentiment, however, fails to account for Canada's health human resources planning errors that create a "pull" and the role of Canadian immigration policy in easing entry for skilled professionals, as well as Canada's obligations under the right to health (i.e. Article 12 of the International Covenant on Economic, Social and Cultural Rights).

A small number of respondents remarked that it would be in Canada's best interest to support bilateral agreements as a means to stem its own brain drain of health care professionals to the United States and other countries. Bilateral agreements could protect Canada against losing any gains it is trying to make in balancing health human resources planning.

#### Reparation payments/restitution to source countries

Respondents were asked for their views on the idea of financial reparation to source countries for training and health system losses from the migration of health professionals, a claim frequently made by SSA governments. The great majority of respondents completely dismissed financial restitution as a viable option. Most respondents felt reparations would be "delicate", "difficult", and even "impossible" to oversee. Some clarified their opposition, explaining that, as there is no explicit public policy to recruit from these countries, Canadians would not want to reimburse for persons they had not "stolen" or "poached" in the first place. In their view, many foreign-trained health care workers were coming to Canada on their own initiative, having sought and been offered jobs prior to immigrating. They expressed concern that Canadians would feel that, since they decided to come to Canada on their own, there would be no reason why Canada should pay another country for their personal decision to migrate "we didn't ask them to come... they decided to come on their own in many cases."

Most respondents also made the point that the rotating door or circulation phenomenon – wherein countries, including Canada, lose health care providers through emigration and gain others through immigration – meant that foreign-trained practitioners in Canada could leave the country. As foreign-trained health care workers were not bonded to Canada, they would be free to migrate to a third country, with Canada having paid their tab for reparations and subsidized their credentialing and retraining. Seeking repayment by the third-party country would be even more difficult, while placing foreign-trained practitioners on bonds upon arrival into Canada was frowned upon.

Many respondents also argued that it was not reasonable or fair to single out the health care workers from the larger problem of the emigration of all types of skilled professionals from SSA countries. This logic would extend reparation for the loss of all skilled professionals who migrate from one country to another, creating a political, economic and accounting problem of such complexity as to be unimaginable in practice.

#### Increased training of auxiliary workers in source countries

Another option gaining traction in international policy debates is that of increasing the training of auxiliary (lower-skilled) workers in source countries, rather than focusing on nurses and physicians [23]. Auxiliary workers would still be of enormous benefit to source countries with serious deficits in health care workers, but would not likely gain entry into Canada or other developed countries on the merit of their training.

Views differed on this option, although the majority indicated strong opposition. Some believed it would result in a less-trained medical force, which could be more detrimental to the country's health care system. Others argued in a similar vein that the fragmentation of the health workforce through the development of ad hoc groupings of subprofessional categories would be somewhat problematic, as it would tend to result in the "de-skilling" of some professionals. One respondent, himself foreign-trained, found the idea verging on racist, arguing that while Western countries would likely continue receiving physicians and nurses from SSA countries (since this option would have no effect on reducing their migration), SSA source countries would be left with inferior health care workers to care for their populations.

Others judged it to be a good idea, since there are typically not enough primary health care workers in many source countries and that such workers, who could provide a great deal of basic treatment, need not be fully-qualified nurses or doctors. As one respondent expressed, "We focus on training the top end to the detriment of putting some of these lower-end supports in place and I think [the latter] would be highly effective."

#### Restrictions on health professional migration from underserved source countries

Interviewees were asked if their organizations would support restricting the migration of health professionals from SSA and other developing countries with severe deficits in health care workers. Restrictions could ostensibly be applied by placing health practitioners seeking to emigrate from underserved source countries on a "low priority or points" list, a situation that actually existed in Canada during the early 1990s, when it was believed there were too many physicians and nurses already practising. All respondents were opposed to this solution, firmly believing in migration as an individual right that should not be limited by place of origin and profession. Instituting such restrictions, they explained, would be "un-Canadian" and "against fundamental Canadian values." They followed up on this view with the argument that, as long as strong push factors in source countries existed, migration would continue. There would also always be a degree of "natural mobility" with health care professionals and others seeking to improve their quality of life.

#### Bonding of health care professionals

Bonding would require graduate health professionals to remain in service in their countries of training upon graduation as a way of repaying the government-funded portion of their education. The bond would likely be for a number of years and/or service in a specific location, and could include a financial penalty if broken. This option could be applied to health care workers both in source countries and Canada.

Respondents felt it was up to source countries to decide whether bonding their health workers would be a suitable and effective way to stem the drain from their countries. While some said they would "support" such arrangements, it was made clear that this was beyond their mandate and ultimately "not our business." In any event, it might well be that bonding of new graduates in source countries would not be useful in stemming migration; statistics show that health care professionals (at least physicians) are older when they migrate to Canada and would already have completed their period of bonding [30].

A number of respondents noted that bonding arrangements are already in effect in Canada. There was general consensus that such arrangements are worthwhile, albeit evidence of bonding effectiveness remains scant [23]. But respondents emphasized that such arrangements should remain optional, with a buy-out clause whereby a penalty could be paid if the bond (contract) terminated early. Others remarked that Canada should consider such bonds to minimize its own brain drain to the United States (or to the United Kingdom); several respondents would like to see pan-Canadian bonding policies in place to reduce interprovincial brain drain.

#### Health system strengthening in source countries

Health systems strengthening in SSA countries would result in far fewer push factors. This option was widely supported by respondents, falling in second place as a preferred measure after improved HHR planning in Canada. However, respondents also quickly noted that, for the most part, this option was largely out of their control and required policy action and involvement of Canada's foreign affairs department, its donor agencies and other multilateral institutions.

Some respondents cited localized examples of how their organizations have already been involved in capacity building, skills training, policy instruction and other initiatives in developing countries. Others emphasized that such efforts should be focused bilaterally (government to government, rather than institution to institution) to strengthen their impact; and that, even under such an arrangement, it would be complex to orchestrate or support inter-institutional exchanges.

As in the case of bonding, numerous respondents expressed that efforts to strengthen health care systems in source countries were matters of their personal concern but had little to do with the mandates of their own organizations. They nonetheless indicated that this policy option could be promoted to, and gain broad political support from, most members or constituents of their organizations. Moreover, increased aid from Canada to the health systems in SSA countries (including but not restricted to source countries) was seen as a more viable means of compensating source countries than reparations. Whether Canadian health development assistance to source countries exceeds estimates of Canada's gains or source countries' losses from health professional migration is still under empirical assessment.

Colloquium participants reconfirmed strong support for the option of strengthening health systems in source countries, stressing this as the option of first call. Many repeatedly argued that push factors are principally at the root of the exodus of health professionals. Since a good number of these factors relate to the working environment (pay, long and stressful hours of work, poor safety, inadequate equipment and materials, absence of promotions), health professionals would continue to migrate regardless of measures taken in recipient countries.

## Conclusion

The unique structure of Canada's health care system makes it particularly difficult to determine where ultimate responsibility to mitigate the health costs incurred by the flow of health professionals from sub-Saharan Africa to Canada lies. It is also the reason why it is difficult to obtain consensus on policy options to reduce reliance on foreign-trained health care workers. Regardless, it is clear that unless Canada and source countries take some action, the brain drain of health care professionals from SSA to Canada will continue. The greater fear is that, as Canada's shortages in physicians and nurses become exacerbated (as predicted), so will the brain drain. Unless measures are adopted, Canada will likely continue to receive significant numbers of health care professionals from sub-Saharan Africa, a region itself so desperate for their skills. So how will change occur?

Most interview and colloquium respondents felt that, in order to diminish the exodus of health professionals from the SSA region, source countries themselves had to tackle the factors pushing their health workers out. On the one hand, several SSA countries have yet to abide by their own Abuja agreement to spend 15% of their national budgets on health; concern over how much of allocated health funding reaches the "front lines" of service delivery persists. While complaining of health professional outflows, several SSA countries have large numbers of unemployed health workers [23], creating an environment in which migration is not only a logical consequence, but perhaps inevitable.

These domestic policy areas demand prompt attention. On the other hand, it is widely accepted that many SSA countries lack the financial capacity themselves to build (or rebuild) sustainable public health systems, and are encumbered by "medium term expenditure frameworks", required by the international financial institutions, that restrict overall public sector salary spending. These problems demand multilateral attention. But while respondents recognized that Canada could do more in this area, aid or other foreign-policy solutions (e.g. with respect to trade or macroeconomic conditionalities) had not yet entered the policy discourse of most of their organizations.

At the same time, respondents felt Canada must reduce pull factors through improved health human resources planning. Severe shortages of physicians and nurses in Canada would only continue to feed Canada's dependency on foreign-trained professionals, the lack of a clear deficit/inflow relationship notwithstanding.

Stakeholder organizations would like to see increases in residencies and medical and nursing school enrolments as well as programmes for retraining and licensing individuals having difficulty obtaining certification in Canada, so that Canadians trained abroad and foreign-trained health professionals in Canada unable to practise can more readily join Canada's under-serviced health workforce. As with SSA source countries, however, many receiving countries – including Canada and the USA – have reasonably large numbers of qualified health professionals not employed in the health sector, particularly in nursing. (Indeed, the number of RNs in the United States not working as nurses is roughly two thirds of the anticipated shortage.)

At least some health workers in receiving countries are being "pushed" out of their profession (albeit not always out of their country) for reasons of overwork, understaffing, stress and shift-work arrangements that make work/life balance hard to maintain [19,21]. Reducing staff shortages in recipient countries, then, entails more than simply increasing numbers of domestic trainees.

Although it was beyond the remit of our study to determine what mix of health workers would be best for any given country or context, the lack of respondents' support for increasing the training of auxiliary health workers in SSA countries is somewhat surprising. There is obvious self-interest on the part of specific health professions to retain monopoly rights over scope of practice, which could extend to taking a dim view of increasing the number of lesser-skilled workers who would constitute a type of professional competition. Just as we cannot infer motives for our respondents' views on the other seven options proposed to them, we cannot assume motives for their generally negative response to this option. However, the caution that – at least in the absence of other interventions to stem the flow of physicians and nurses – this option could create the perverse outcome of a singularly lesser-skilled health system in SSA deserves attention. At the same time, many of the health interventions leading to rapid declines in infant, child and maternal mortality could be undertaken by adequately trained, salaried and supported health workers functioning below nursing skill-level, provided there is access to higher-skilled services, particularly in emergency situations.

Similarly, the lack of support for bilateral agreements (whether voluntary or mandatory), at least on the grounds of no evidence of success, could be challenged by more recent data from the United Kingdom. While its National Health Service's (NHS) code on ethical recruitment failed in its first two years to stem the flow of nurses from SSA, the total numbers of foreign-trained nurses began to decline in 2004/05 while enrolments for domestic training that year were 67% higher than in 1996/97 [31]. (This nonetheless will have the NHS continue to rely on foreign-trained nurses to staff about 25% of its positions, for which it is looking increasingly to recruits from India and the Philippines [32].)

Responding to several criticisms from the South African government regarding Canada's high ratio of South African-trained physicians, the Canadian federal government announced that it was exploring the potential of developing a Memorandum of Understanding with South Africa (following the lead of the United Kingdom), and undertook a scoping mission to South Africa in late 2005, after our interviews had been completed.

Interestingly, the human rights dimension of the global flow of HHR was largely absent from the respondents' discussion of policy options. This can be attributed in part to a lack of specific questioning on this topic, although the semistructured interview schedule did prompt respondents on human rights in many instances. Perhaps reflecting Canadian interests, the right to migrate (more accurately, the right to seek migration, since there is no reciprocating obligation on countries to accept all who arrive at their borders) was frequently referred to, while the right to health of communities affected by the drain of HHR in SSA was not. When raised as a topic, most respondents were generally favourable to the idea that the right to health, and commitments to such international targets as those of the Millennium Development Goals, obliged Canada to act differently with respect to the global HHR crisis.

We finally need to caution readers about inferring too much from this one study. Our sample, while purposive, was small. Our data sets, while as complete as we could obtain, were still imprecise, especially regarding how long foreign-trained health workers remained in Canada, and where they went when they left. Our understanding of the actual recruitment practices of Canadian regional health authorities or other health system employers is very limited. Our estimates of the financial costs and benefits of migration, to both source and receiving countries, are nascent. These are all areas where a new set of studies is commencing.

In closing, there is indication of some small steps being taken in Canada to address the global HHR problem, but there remains little public, political or even professional awareness of the issue as it affects SSA countries. Moreover, Canadian federal and provincial governments are simultaneously facilitating greater immigration of foreign-trained health care workers, through the adoption of measures such as the Provincial Nominee Program and the new immigration points system favourable towards professionals such as physicians and nurses. Advertisements for physicians for rural Canadian posts continue to proliferate in southern African medical journals. Both Canadian and SSA parties to the "brain drain" have yet to give the issue the political and policy attention it warrants, albeit momentum is slowly building in that direction.

## Competing interests

The author(s) declare that they have no competing interests.

## Authors' contributions

RL was principal investigator for this research and conducted a majority of the interviews. NK conducted a number of the interviews, and both CP and NK undertook most of the qualitative data analysis. CP, NK and RL wrote the first full draft of this article and all three contributed to revisions.

**Figure 4:**
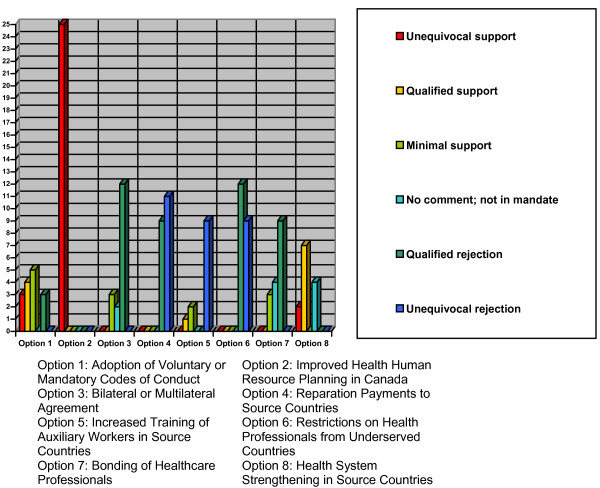
Survey respondents' support for policy options to mitigate the effects of, or reduce, the brain drain of health professionals to Canada.
